# Sentiments about Mental Health on Twitter—Before and during the COVID-19 Pandemic

**DOI:** 10.3390/healthcare11212893

**Published:** 2023-11-03

**Authors:** Felix Beierle, Rüdiger Pryss, Akiko Aizawa

**Affiliations:** 1 National Institute of Informatics, Tokyo 101-8430, Japan; aizawa@nii.ac.jp; 2 Institute of Clinical Epidemiology and Biometry (ICE-B), University of Würzburg, 97074 Würzburg, Germany; ruediger.pryss@uni-wuerzburg.de

**Keywords:** COVID-19, coronavirus, public health, sentiment analysis, topic modeling, machine learning

## Abstract

During the COVID-19 pandemic, the novel coronavirus had an impact not only on public health but also on the mental health of the population. Public sentiment on mental health and depression is often captured only in small, survey-based studies, while work based on Twitter data often only looks at the period during the pandemic and does not make comparisons with the pre-pandemic situation. We collected tweets that included the hashtags #MentalHealth and #Depression from before and during the pandemic (8.5 months each). We used LDA (Latent Dirichlet Allocation) for topic modeling and LIWC, VADER, and NRC for sentiment analysis. We used three machine-learning classifiers to seek evidence regarding an automatically detectable change in tweets *before* vs. *during* the pandemic: (1) based on TF-IDF values, (2) based on the values from the sentiment libraries, (3) based on tweet content (deep-learning BERT classifier). Topic modeling revealed that Twitter users who explicitly used the hashtags #Depression and especially #MentalHealth did so to raise awareness. We observed an overall positive sentiment, and in tough times such as during the COVID-19 pandemic, tweets with #MentalHealth were often associated with gratitude. Among the three classification approaches, the BERT classifier showed the best performance, with an accuracy of 81% for #MentalHealth and 79% for #Depression. Although the data may have come from users familiar with mental health, these findings can help gauge public sentiment on the topic. The combination of (1) sentiment analysis, (2) topic modeling, and (3) tweet classification with machine learning proved useful in gaining comprehensive insight into public sentiment and could be applied to other data sources and topics.

## 1. Introduction

Especially at the beginning of the COVID-19 pandemic, there was a significant amount of uncertainty regarding the new coronavirus SARS-CoV-2. In addition, several countries started to implement strict social distancing measures such as lockdowns, work from home, etc., in March 2020. Both the uncertainty about the virus itself and the social distancing measures could have affected the mental health of the population. Depression is one of the most prevalent global mental health issues and accounts for the largest share of the World’s burden of disease [1].

There has been some prior research on the public perception or public sentiments about the topic of mental health during the pandemic. It is typically based on smaller-scale data collected via surveys [2,3,4,5,6,7,8,9,10,11] or apps [12,13,14]. Analyzing the sentiments of a larger segment of the population regarding mental health can help public health officials gauge the direction of overall trends. In particular, comparing data during the pandemic with pre-pandemic data may contribute to a more complete understanding of changes in the public perceptions of mental health and depression. The assessment of public opinion may then potentially help to assess the state of health care systems and the needs and expectations of the public.

While there are several publications on the analysis of Twitter (re-branded to X in July 2023) data related to the COVID-19 pandemic, which we will discuss below, most of them do not focus on explicit sentiments regarding mental health; moreover, most lack a comparison between the period before and the period during the pandemic.

The research question we specifically answer is: With which sentiments are Twitter users tweeting about depression and mental health and how did these sentiments change during the COVID-19 pandemic? In the following, we analyze tweets containing #MentalHealth and #Depression by assessing (1) sentiments over time, (2) topic modeling, and (3) a machine-learning-based binary classification of tweets *before* and *during* the pandemic, with three distinct approaches. Our main contributions are twofold: (a) the collection and analysis of a large dataset of mental-health-related tweets spanning a long time period (17 months total, 8.5 month before and 8.5 during the pandemic); (b) reporting on the usefulness of the combination of the three methods (sentiment analysis, topic modeling, tweet classification).

This paper is structured as follows. In Section 2, we highlight related work. In Section 3, we detail the data collection from Twitter as well as the methods used in our work. We present and visualize our results in Section 4. In Section 5, we discuss our findings and point out limitations, before we conclude in Section 6.

## 2. Related Work

There are several papers which examine social media in general or tweets in particular in the context of the COVID-19 pandemic. For example, Gianfredi et al. examined published work on what online-users’ behavior can tell us about mental health outcomes during the pandemic [15]. Some of the reviewed studies used data from Twitter, while other sources included Google Trends, Google Search, and Reddit. Gianfredi et al. reviewed 19 papers and found an overall increase in searches for terms such as anxiety, depression, and stress.

Most of the specific related work regarding data from Twitter about the topic of mental health and the COVID-19 pandemic looks at the period of the pandemic exclusively, and mostly at short timeframes. Boon-Itt and Skunkan conducted sentiment analysis and topic modeling for tweets from December 2019 to March 2020 and reported an overall negative outlook toward COVID-19 [16]. They used keywords, TF-IDF (Term Frequency-Inverse Document Frequency), the sentiment library NRC Word-Emotion Association Lexicon, and LDA for topic modeling. Lwin et al. analyzed tweets from January to April 2020 and reported a shift from *fear* towards *anger* as the primary emotion associated with tweets about the novel coronavirus [17]. They based their results on a tool called *CrystalFeel*. For tweets between January 2020 and April 2020, Mathur et al. reported an almost equal amount of positive and negative sentiment, and *trust* and *fear* as the most frequent emotions recognized, based on results from the NRC sentiment library [18]. Manguri et al. analyzed tweets from a single week in April 2020 and reported observing more positive than negative sentiment, with mostly neutral tweets [19]. They used the TextBlob library for sentiment analysis. Xue et al. analyzed tweets from March to April 2020 and identified 13 topics with LDA, which were all connected to a mix of sentiments of *trust*, *anger*, and *fear* [20]. Valdez et al. analyzed tweets from the period of January to April 2020 and reported an overall positive trend [21]. Additionally, they analyzed the individual timelines of more than 300,000 users from major US cities and found that social media use was peaking during stay-at-home mandates, and that for the users analyzed the sentiments of their tweets trended negatively. They also used LDA, and additionally the VADER sentiment library (Valence Aware Dictionary for sEntiment Reasoning). Sarirete investigated tweets from December 2020 and January 2021 regarding COVID vaccines specifically and reported mixed sentiments, with negative emotions dominating [22]. Keywords and TF-IDF were used for this analysis. Looking at the work cited in this paragraph, the differences in sentiments likely depend on the specific time frame in which the tweets were analyzed, as well as the specific search queries for obtaining the tweets.

Other related work investigated longer timeframes or used methods other than sentiment analysis and topic modeling. Cohrdes et al. analyzed the tweet contents from German Twitter users from the first half of 2020 and reported depressive symptoms, signaled most often by *fatigue* and *loss of energy* [23]. A proprietary linguistic tool was used for the analysis. Koh and Liew analyzed the regional differences in tweets regarding loneliness during May and June 2020, showing, for example, how European Twitter users tweeted less about the community impact of COVID-19 compared to North American Twitter users [24]. Their results were based on the *stm* R package for topic modeling. Zhang et al. analyzed the demographical differences in tweets from March 2020 to January 2021 and reported that white users and male users showed higher concerns regarding mental health [25]. They used LDA for their analysis. Zhang et al. collected data specifically from users with depression [26]. They trained a model combining deep learning (a transformer model) and psychological text features and reported 78.9% accuracy for detecting depressed users.

Some related work has looked into the differences in tweets before and after the pandemic. In their short paper, Guntuku et al. reported on a dashboard they developed for gaining insights regarding tweets about mental health during the pandemic [27]. In the paper, they compared a two-months timeframe from March to May 2020 to the same timeframe in 2019 and reported observing lower mental health and higher *stress*, *anxiety*, and *loneliness* in 2020. They used pre-trained machine-learning models not specified in detail. El-Gayar et al. collected data from 2017 to 2021 and reported an increase in mental-health-related tweets after COVID-19 started spreading [28]. For their analysis, they manually labeled some data and algorithmically labeled the rest of the tweets. Saha et al. collected 60 million tweets from a two-month time window between March and May 2020, as well as 40 million tweets from the same time window in 2019 [29]. They concluded that they observed significant increases of mental health symptomatic expressions in 2020. Their experiments were based on classifiers trained on data from Reddit. Ng et al. analyzed 68,345 tweets about loneliness posted by organizations between 2012 and 2022 [30]. Using BERTopic for topic modeling, they reported that a significant proportion of tweets were centered on the impact of COVID-19 and loneliness.

Overall, most related work examines rather short periods of Twitter data, usually focusing on the onset of the pandemic. Few of the works compare the data with tweets before the pandemic. While the related works collected data using different strategies, we will examine tweets that were specifically tagged by the tweet authors with a hashtag related to #MentalHealth or #Depression, which will help us gain insights into public perceptions of this issue. The review of the related work shows that sentiment analysis and topic modeling are established methods for analyzing Twitter data. Common approaches are LDA for topic modeling and the use of sentiment libraries such as NRC for sentiment analysis. We argue that our machine-learning-based classification can provide additional benefits for assessing changes between two time periods, specifically by giving evidence about the extent to which a change in sentiments or tweet contents is automatically detectable.

## 3. Materials and Methods

In this section, we describe the data collection, data processing, and the applied techniques of sentiment analysis, topic modeling, and classification of tweets.

### 3.1. Data Collection

We collected all original tweets (i.e., we excluded retweets) containing #MentalHealth or #Depression between April and August 2021 via the Twitter API. When only considering English tweets, as determined by Twitter metadata, from all tweets from 2019 and 2020, this leaves 92.34% of tweets containing #MentalHealth and 84.94% of the tweets containing #Depression. To account for seasonal effects, we used the same time window before and after the pandemic. As we wanted to investigate the effects of the pandemic, we needed two datasets that were clearly separated, i.e., that were not overlapping or ambiguous in terms of belonging in either category. The first cases of SARS-CoV-2 became public at the end of November 2019. Any Twitter user tweeting before 1 December 2019 would not have the COVID-19 pandemic in mind. The WHO declared the COVID-19 pandemic on 11 March 2020 (https://www.who.int/europe/emergencies/situations/covid-19, accessed on 12 September 2023). Several countries started implementing social distancing measures, trying to slow down the spread of the virus. It is safe to assume that any Twitter user tweeting after 11 March 2020 was aware of the pandemic. Therefore, the time windows we chose for the period before and during the pandemic, which we consider robust, were 11 March to 1 December for 2019 and 2020.

Looking into the number of tweets per day, we observed outliers, i.e., many more tweets than the average amount on world mental health day, 10 October. Additionally, we found an outlier after 14 June 2020, with almost 17 times the median number of tweets, with #Depression. Investigating the other hashtags used in combination, it turned out that those tweets were about the Indian actor Sushant Singh Rajput, who committed suicide on 14 June 2020 and is said to have suffered from depression. In order to avoid a strong bias toward this singular event, we removed the tweets related to this event, as identified by hashtags, and sub-sampled the remaining tweets to fit the median of the other days.

Table 1 shows the resulting datasets that we used in our analysis. After the described pre-processing, D19 and D20 are almost identical in size. The number of tweets containing #MentalHealth increased from 2019 to 2020 by more than 30%, marking M20 larger than M19. For our machine-learning-based tweet classification, we wanted to have two datasets of equal size. We sub-sampled M20 and created M20S, which has the same size as M19. In the following, we will always specify which dataset is being used.

In pre-processing datasets D19, D20, and M20S, we addressed three elements of tweets: (a) *mentions*, i.e., links to other Twitter accounts, (b) *URLs*, i.e., links to websites, and (c) *hashtags*, i.e., links to other tweets with the same hashtag. We removed mentions and URLs, as they typically did not convey textual information. We removed the ‘#’ symbol in front of hashtags, as hashtags might convey textual content and are sometimes used like words; for example, ‘I am suffering from #depression’.

### 3.2. Sentiment Libraries

Our analysis of the related work in Section 2 has shown that the use of sentiment libraries is a common approach, with, for example, NRC and VADER being used. In our work, we use three sentiment libraries to annotate each tweet: (a) LIWC (Linguistic Inquiry and Word Count) [31,32], (b) the NRC Word-Emotion Association Lexicon [33,34], and (c) VADER (Valence Aware Dictionary for sEntiment Reasoning) [35]. LIWC was developed by researchers from different fields, including psychology, and captures the percentage of words that reflect different emotions, concerns, etc. VADER was developed via crowdsourcing and is “specifically attuned to sentiments expressed in social media”. (https://github.com/cjhutto/vaderSentiment, accessed on 12 September 2023). It yielded values for *negative*, *positive*, and *neutral* sentiments. NRC was developed via crowdsourcing and yielded values for eight emotions (anger, anticipation, disgust, fear, joy, sadness, surprise, trust) and for *positive* and *negative* sentiments (https://saifmohammad.com/WebPages/NRC-Emotion-Lexicon.htm, accessed on 12 September 2023). In addition to the sentiment annotation, we mapped the time of day of the tweet to a float value between 0 and 1. We then had a total of 112 features for each tweet. We used these features to describe the sentiments with which users tweet about depression and mental health, as well as for one of our three machine-learning classifiers.

### 3.3. Topic Modeling with Latent Dirichlet Allocation

Most of the related work covered in Section 2 that conducted topic modeling did so by applying LDA. LDA is a generative probabilistic model that can find topics in text corpora. LDA finds topics based on sets of terms (words or phrases) and determines the relevancy of each document (tweet in our case) for each discovered topic. For more details, refer to [36]. We applied LDA for describing the topics present in datasets D19, D20, M19, and M20. We first used Gensim for pre-processing [37]. We applied lemmatization on all texts, i.e., using the base form of words, reducing the number of inflectional and derivational forms, with spaCy (https://spacy.io/, accessed on 12 September 2023). After removing stopwords, we used scikit-learn (https://scikit-learn.org/, 12 September 2023) for a cross-validated grid-search for finding the number of topics in our datasets, and we reported the most common keywords in each topic.

### 3.4. Classification

For classification, we used three different approaches on datasets D19 and D20, as well as datasets M19 and M20S. The general idea was that we wanted to see if a machine classifier could classify if a given #Depression or #MentalHealth tweet was from *before* or *during* the pandemic. If a classifier was able to classify correctly, it was evidence for a change in the public sentiment about these topics during the COVID-19 pandemic. The three classifiers cover different aspects: the TF-IDF classifier serves as a baseline and considers the frequencies of important words. The sentiment-libraries-based classifier consists of features calculated based on sentiment libraries created by human experts. The BERT classifier is a deep-learning black-box approach that processes whole tweets instead of metadata.

#### 3.4.1. Based on TF-IDF

The first classifier we implemented as a baseline was based on TF-IDF (Term Frequency-Inverse Document Frequency). TF-IDF is a statistic that indicates how important a word is in a corpus [38]. After using the NLTK SnowballStemmer (https://www.nltk.org/api/nltk.stem.snowball.html, accessed on 12 September 2023) for obtaining the stems of each word, we calculated the TF-IDF values for unigrams and bigrams. These values were fed into the pipeline described in the following.

Having tabular data, we sought to apply traditional tree-based machine-learning approaches. We did not expect different classifiers to perform vastly differently and decided to use LGBM (Light Gradient Boosting Machine) because it is known for high performance and high efficiency [39]. LGBM is a gradient boosting approach that builds a classifier based on an ensemble of weak classifiers. We classified the data into classes 0 (*before* the pandemic) and 1 (*during* the pandemic). We used a train/test split of 80%/20% and conducted a hyperparameter search via a cross-validated grid search while optimizing for accuracy. For the evaluation of our models, the main metric we used was accuracy, which is the percentage of correctly classified tweets. As the classification is binary and because D19 and D20, as well as M19 and M20S, are of equal sizes (see Table 1), accuracy is an appropriate metric to be applied. Random guessing would yield an accuracy of 0.5. Additionally, we reported the area under the receiver operating characteristic (ROC) curve. The ROC curve plots the true positive rate against the false positive rate at various thresholds. The area under the curve (AUC) is often reported for classification problems as an indication of the model’s performance. The range is 0 to 1, with 0.5 indicating random predictions. In addition, we also reported precision (percentage of tweets classified as *during* the pandemic that were correctly classified; range 0 to 1), recall (fraction of all *during* tweets that were correctly classified; range 0 to1), F1 score (harmonic mean of precision and recall; range 0 to 1), and MCC (Matthew’s Correlation Coefficient, range −1 to 1).

#### 3.4.2. Based on Sentiment Libraries

The second classifier we implemented utilized the 112 features from the three sentiment libraries. These features were fed into a similar pipeline as with the TF-IDF-based approach, using LGBM, with a 80%/20% train/test split and a cross-validated grid search for hyperparameters. For evaluation, we again used accuracy as the main metric and reported ROC-AUC, precision, recall, F1, and MCC.

#### 3.4.3. Based on BERT

Our last approach for classification takes as input the whole text of the tweets instead of computed values such as sentiment or TF-IDF values. BERT (Bidirectional Encoder Representations from Transformers) is a transformer-based machine-learning approach developed by Google and is widely applied in NLP (natural language processing). The wide adoption of BERT in NLP led us to choosing BERT instead of older deep-learning approaches based on recurrent neural networks. BERT models pre-trained on vast amounts of data can capture complex contextual relationships in language. To capture these relationships, BERT builds on multiple layers of self-attention heads. For more details, refer to [40]. We used the pre-trained BERT model ‘bert-base-cased’ and the Huggingface library (https://huggingface.co/bert-base-cased, accessed on 12 September 2023) for processing the full tweet texts and classifying between before and during the pandemic with PyTorch [41]. The train/validation/test split was 80%/10%/10%. We used the same evaluation methods as with the other classifications: accuracy and area under the ROC curve.

## 4. Results

In this section, we present the results. We report the statistics of the dataset before providing the results from the analysis of the sentiment libraries, the topic modeling, and tweet classification.

### 4.1. Data Statistics

Using Python, we first look into the datasets D19, D20, M19, and M20. There are some metadata provided by Twitter regarding the tweets. Tweets can be marked as ‘possibly sensitive’. This is the case for between 0.52% and 0.84% of the tweets in the four datasets. Similarly, for the ‘restricted in country’ flag, we do not see significant changes between the datasets; there are always fewer than 50 tweets flagged. Location information within the ‘places.country’ field is only available for a fraction of the tweets. This fraction is between 2.56% and 4.12% for D19, D20, M19, and M20.

There are several clients that can be used to publish tweets. The most used ones are the ones provided by Twitter themselves. These include (a) ‘Twitter Web App/Client’ and (b) ‘Twitter for iPhone/Android’. (a) is a client in the browser and (b) is a mobile client. Note that (a) could be a browser on a smartphone, though we assume this is unlikely. Overall, these clients, (a) and (b), account for 52% to 60% of all the tweets. Mobile clients are used more than browser-based clients and we do not observe significant changes between the 2019 and 2020 datasets.

In Table 2 and Table 3, we present the most frequent hashtags in D19 and D20. In Table 3, we marked those hashtags that are related to the COVID-19 pandemic. In Table 4 and Table 5, we present the most frequent hashtags for M19 and M20. Again, we highlight the COVID-19-related hashtags.

### 4.2. Sentiment Analysis Results

**Figure 1:**
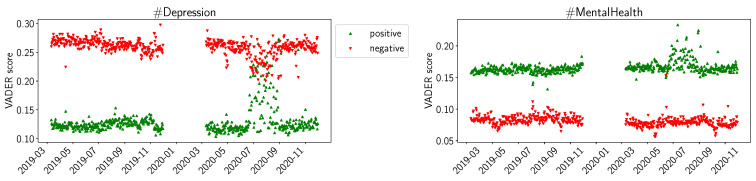
VADER average daily sentiments (positive, negative) for D19, D20, M19, M20.

In Figure 1, we present the trends we can observe from the values calculated with the VADER sentiment library. We plotted the daily average *positive* and *negative* sentiment as indicated by VADER. We can observe that, overall, for #Depression, the negative sentiment is higher than the positive one, and for #MentalHealth the opposite is true. For #Depression, we note a trend during the summer of 2020 (left hand side of Figure 1): the red downward triangles indicate that the daily average negative sentiment is decreased, while the green upward triangles indicate that the daily average positive sentiment is increased. For #MentalHealth (right hand side of Figure 1), we observe the same trend, but only for the positive sentiment.

**Figure 2:**
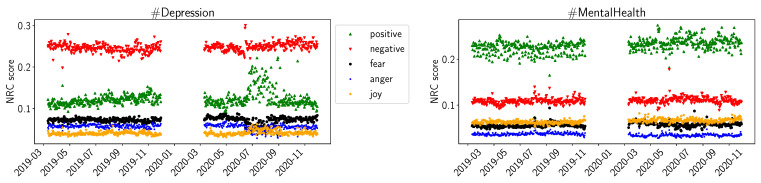
NRC average daily sentiments (positive, negative, fear, anger, joy) for D19, D20, M19, M20.

In Figure 2, we show the results of the NRC sentiment library. We plotted the average daily values for *positive* and *negative* sentiment, as well as for *fear*, *anger*, and *joy*. For #Depression, we observe the same trend of increasing daily average scores during the summer of 2020. However, compared to VADER, NRC did not indicate a change in the *negative* sentiments. In the #MentalHealth datasets, we do not observe significant changes in daily average scores for *positive* or *negative* sentiments. The scores for *fear*, *anger*, and *joy* seem somewhat constant over time for #MentalHealth. For #Depression, *fear* and *anger* seem to decrease slightly during the summer of 2020, while *joy* seems to increase slightly in the same timeframe.

**Figure 3:**
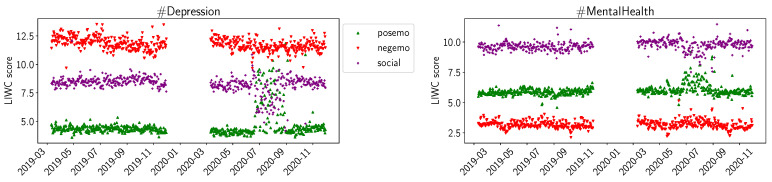
LIWC average daily sentiments (positive emotion, negative emotion, social) for D19, D20, M19, M20.

**Figure 4:**
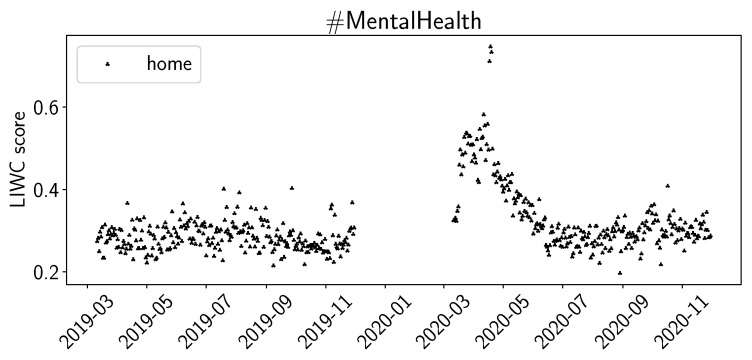
LIWC average daily sentiment (home) for M19, M20.

For the sentiment scores of LIWC, we plot *posemo* (positive emotion), *negemo* (negative emotion), and *social* in Figure 3. For the summer of 2020, we observe increased scores for *posemo* in both #Depression and #MentalHealth, while we do not observe significant changes for *negemo*. For *social*, we observe a decrease for summer of 2020 in #Depression and a slight decrease in #MentalHealth. We observed similar trends for the summer of 2020 for the LIWC values for *work*, *achieve*, and to a lesser extend, for *leisure* and *reward*. In Figure 4, we plot the LIWC values for *home* for #MentalHealth. The daily averages increase by a factor of 2 to 3 at the beginning of the pandemic and, over time, decrease again to previous levels by June 2020. We can observe a similar trend in #Depression, but the increase at the beginning of the pandemic is not as large.

### 4.3. Topic Modeling Results

In Table 6, we present the results for topic modeling with LDA. Judging by the keywords, the common themes across both hashtags are *awareness* (e.g., referring to either ‘mental health’ or ‘depression’ explicitly), *support* (e.g., ‘mindfulness’, ‘treatment’, ‘service’, ‘support’), *symptoms* (e.g., ‘anxiety’, ‘stress’, ‘suicide’, ‘symptom’), and *gratitude* (e.g., ‘thank’, ‘joy’, ‘love’, ‘good’). During 2020, we see references to the pandemic situation, specifically COVID and *social distancing* (‘quarantine’, ‘lockdown’). Note that there is some overlap in the themes between the topics. We observe that *gratitude* increased for #MentalHealth from 2019 to 2020. While COVID and *social distancing* seems to mostly be related to the third topic for #Depression in 2020, the pandemic permeates all three topics for #MentalHealth.

### 4.4. Classification Results

In Table 7, we present the results of the three classification approaches. The main evaluation metric of accuracy, as well as all other metrics, show for both cases (#Depression and #MentalHealth) that BERT performs best. The TF-IDF-based baseline classifier performs better than the classifier based on the features of the sentiment libraries. While the TF-IDF classifier seems to perform very similarly on the datasets of both hashtags (74% accuracy for both), the sentiment-library-based classifier performs a little better on the #Depression dataset (72% vs. 68% accuracy). The BERT classifier, on the other hand, performs a little better on the #MentalHealth dataset (79% vs. 81% accuracy).

## 5. Discussion

While we do not see an increase in tweets for #Depression, we observe an increase of 31% for tweets in M20 compared to M19 (see Table 1), and there are 38% more unique users using the hashtag. It seems as though there is an increase in public interest in the topic of mental health. There could be several reasons for this trend, either related to the coronavirus pandemic or independent of it.

Looking at the most frequent hashtags (Table 2, Table 3, Table 4 and Table 5), we observe an overlap between the sets (#Depression in M19/M20 and #MentalHealth in D19/D20 and, e.g., #anxiety, #mentalhealthawareness). Looking at the hashtags, #Depression seems to be more focused on symptoms or negative experiences (#ptsd, #stress, #addiction, #suicide), while #MentalHealth contains more positive words (#wellbeing, #selfcare, #wellness). Maybe the hashtag #MentalHealth is more associated with general discussion about the topic, including the positive aspects of mental health, and #Depression is more associated with the negative experiences of having depression. Regarding the pandemic, #lockdown is the most frequent hashtags in D20, present in only 5% of the tweets. In M20, #COVID19 is the most frequent hashtag overall, present in 9% of all tweets. This can be considered evidence that the pandemic is one factor driving the discussion about mental health.

The values for positive and negative sentiments in VADER (Figure 1) and NRC (Figure 2), and the sentiments for positive and negative emotion in LIWC (Figure 3), support our interpretation that #Depression is used for more negative sentiments while #MentalHealth is associated with more positive aspects; negative sentiment is higher than positive sentiment for #Depression, and the opposite is true for #MentalHealth. There is some overall consistency between the three sentiment libraries, while the change in the summer trend is not equally visible. This is likely due to the way the libraries work internally. The change in the summer of 2020 could be related to the change in the overall pandemic situation. Maybe people became more used to it, or lockdown restrictions were loosened over the summer; going out was easier in warmer weather as the probability of infection was considered lower while being outdoors. The LIWC sentiment score for *home* (Figure 4) is likely linked to the initial lockdown measures and increased time spent at home and in home office. In their analysis of a large COVID-19-related tweet dataset, Arpaci et al. also found that “home” is a common word used between March and April 2020 [42]. We interpret the comparatively high scores for positive sentiments to signal support and raising awareness by the users using the hashtags, especially #MentalHealth. The rather stable sentiment values outside the beginning of the pandemic might indicate a rather quick return to baseline. It seems as though the pandemic might not have lastingly changed the sentiments with which Twitter users tweet about mental health topics.

Looking at the topics identified by LDA and their themes (Table 6), we note that for #Depression, COVID does not play such a big role; general *awareness* and *support* tweets make up roughly the same amounts in 2019 and 2020. For #Depression, in 2020, COVID is mentioned together with symptom-related tweets. For #MentalHealth, COVID and social distancing have a stronger influence on tweet content, with COVID- and social-distancing-related terms showing up in all of the three identified topics in M20. The largest topic in M20 contains positive words associated with *gratitude*, such as *love*, *good*, or *thank*. This supports the observation that average positive sentiment is higher in 2020 compared to 2019. For the beginning of the pandemic, when considering COVID-19 tweets in general, Arpaci et al. report that negative and fear-related words showed up in relatively high frequencies [43]. We suspect that our findings are different because the subset of users specifically tweeting about mental-health-related topics seems to focus on support rather than on fear. There is some psychological work supporting this interpretation. Ntontis and Rocha report in [44] that in crises such as the COVID-19 pandemic, people can show solidarity based on shared social identity. Maybe the user group of Twitter users tweeting about mental health had such a shared identity, showing solidarity in the form of gratitude and mutual support.

After using sentiment libraries to look into the properties of the tweets in the datasets, we used machine learning to see to what extent we could automatically classify tweets between *before* and *during* the pandemic. The model based on the values of the sentiment libraries performs better than guessing, but not significantly (see Table 7). Looking at the sentiment values in the figures in this paper, we can see why this is likely the case: overall, most of the average values are somewhat constant over time, making it hard for the algorithm to distinguish between the two classes. The TF-IDF-based baseline classifier performs slightly better. Any word specifically associated with the pandemic, such as “COVID”, “quarantine”, or “lockdown”, will only occur in D20 and M20s compared to D19 and M19, likely driving the ability of the algorithm to distinguish between *before* and *during* the pandemic. The BERT model takes the whole tweet text for classification and performs slightly better than the other two approaches. The classification is still not perfect; likely many tweets use words and cover topics that are not bound to specific events and not related to the pandemic. We conducted the classification for single tweets. If we classified by day, i.e., taking all tweets from one calendar day and classify on that level, we might see an increase in accuracy.

### Limitations

There are some limitations to consider. The Twitter users that decide to specifically tweet about the topics of mental health and depression by explicitly using the related hashtags might not be the ones being affected by problems with mental health or depression. Furthermore, we cannot directly infer a general public sentiment from our study. We did not take a sample from the general public but from a subset of Twitter users. However, with up to 300,000 unique users (see Table 1), the sample size is very large, and trends regarding the sentiments of the general population might be reflected in those of the Twitter users. While we observed changes in the tweets before vs. during the pandemic, these changes might not only be influenced by the pandemic but also by other changes in society. Choosing other time periods to conduct our study could yield different results, as could conducting the same analyses for different languages.

## 6. Conclusions

In summary, we found that Twitter users using the hashtags #Depression and especially #MentalHealth are primarily interested in raising awareness of the topic. We observe more positive than negative sentiment for #MentalHealth tweets, an overall increase in related tweets, and an increase in positive sentiment in related tweets. The COVID-19 pandemic runs through all three detected topics of tweets about #MentalHealth. The pandemic influenced public discussion of mental health issues. Notably, the deep-learning-based BERT model can distinguish between tweets from before and during the pandemic, supporting our interpretations of changes in tweet content during the pandemic. Overall, Twitter users used the pandemic and its difficult times—with concerns about the novel virus and associated social consequences such as social distancing—to raise awareness of mental health and to express gratitude.

Twitter allows us to look into the past and observe changes in sentiment on topics over time, as well as understand the influence of specific events. Our approach of using sentiment libraries, topic modeling, and machine-learning-based classification of tweets has proven useful in gaining comprehensive insights. Each technique helps us to understand the content of tweets, changes over time, and the severity of differences between two time periods. Public health officials can use such insights and the techniques presented to analyze and assess public discourse on specific topics. Future work includes applying newer approaches for sentiment analysis that could improve or extend our findings, for example, sentiment analysis based on GRUs [45]. Additionally, future work will include applying our approach to data from other sources such as Reddit or Facebook.

## Figures and Tables

**Table 1:** Datasets containing #Depression and #MentalHealth.

Dataset	Hashtag	Year	No. of Tweets	No. of Unique Users
D19	#Depression	2019	279,900	74,964
D20	#Depression	2020	276,830	78,888
M19	#MentalHealth	2019	995,770	219,119
M20	#MentalHealth	2020	1,304,112	302,496
M20S	#MentalHealth	2020	995,770	254,558

Timeframe for each dataset: 11 March to 1 December. M20S is a sub-sampled subset of M20 that matches the number of tweets of M19.

**Table 2:** Most frequent hashtags in D19.

Hashtag	Count	
#anxiety	96,888	(35%)
#mentalhealth	75,433	(27%)
#mentalhealthawareness	16,347	(6%)
#ptsd	15,244	(5%)
#stress	13,783	(5%)
#addiction	13,583	(5%)
#mentalillness	13,391	(5%)
#mindfulness	13,269	(5%)
#love	12,720	(5%)
#suicide	12,213	(4%)
#onlinetherapy	10,477	(4%)
#health	10,180	(4%)
#jesus	10,129	(4%)

**Table 3:** Most frequent hashtags in D20.

Hashtag	Count	
#anxiety	94,021	(34%)
#mentalhealth	93,272	(34%)
#life	19,595	(7%)
#mentalhealthawareness	17,607	(6%)
#motivation	16,328	(6%)
#meditation	15,233	(6%)
#stress	15,201	(5%)
#lockdown	15,013	(5%)
#ptsd	14,871	(5%)
#COVID19	14,221	(5%)
#quarantine	13,662	(5%)
#stressrelief	13,155	(5%)
#socialdistancing	13,045	(5%)

**Table 4:** Most frequent hashtags in M19.

Hashtag	Count	
#depression	74,901	(8%)
#anxiety	65,608	(7%)
#mentalhealthawareness	59,298	(6%)
#health	42,095	(4%)
#wellbeing	38,971	(4%)
#mentalillness	33,477	(3%)
#psychology	32,050	(3%)
#mentalhealthmatters	30,496	(3%)
#selfcare	30,196	(3%)
#mindfulness	29,012	(3%)
#wellness	28,768	(3%)
#love	19,411	(2%)
#therapy	18,919	(2%)

**Table 5:** Most frequent hashtags in M20.

Hashtag	Count	
#COVID19	112,053	(9%)
#depression	97,113	(7%)
#anxiety	84,284	(6%)
#mentalhealthawareness	81,838	(6%)
#wellbeing	68,717	(5%)
#mentalhealthmatters	62,132	(5%)
#selfcare	54,558	(4%)
#health	46,274	(4%)
#mindfulness	45,045	(3%)
#coronavirus	43,845	(3%)
#wellness	37,226	(3%)
#love	35,380	(3%)
#motivation	32,465	(2%)

**Table 6:** Topics identified by LDA (Latent Dirichlet Allocation).

Dataset	Percentage	Keywords	Theme(s)
D19	44.0%	mentalhealth, love, anxiety, feel, day, life, today, just, make, prayer, help	awareness
	34.7%	anxiety, therapy, mindfulness, online, skype, &, thank, mentalhealth, help, visit, learn	support
	21.3%	anxiety, health, mental, mentalhealth, stress, &, help, people, symptom, treatment, suicide	symptoms
D20	50.3%	mentalhealth, anxiety, feel, help, just, day, love, &, know, life, time	awareness
	31.4%	anxiety, therapy, mindfulness, online, stress, skype, learn, treatment, addiction, visit, &	support
	18.3%	life, anxiety, mentalhealth, COVID, lockdown, motivation, job, health, trend, quarantine, joy	symptoms, COVID, social distancing
M19	41.1%	health, mental, &, support, help, work, child, people, need, issue, service	awareness
	33.8%	anxiety, depression, life, stress, psychology, therapy, love, mindfulness, blog, new, self	support
	25.1%	thank, make, late, day, help, just, time, feel, good, health, talk	gratitude
M20	38.1%	day, love, life, today, stay, time, mindfulness, good, thank, late, lockdown	gratitude, social distancing
	33.1%	anxiety, depression, feel, help, stress, people, know, time, COVID, need, &	awareness, symptoms, COVID
	28.8%	health, mental, support, &, COVID, help, need, time, work, people, care	support, COVID

**Table 7:** Classification results from the three tested classifiers.

Datasets	Classifier	Accuracy	ROC AUC	Precision	Recall	F1	MCC
D19, D20	TF-IDF LGBM	0.745	0.845	0.756	0.719	0.737	0.490
D19, D20	SenLib LGBM	0.721	0.821	0.726	0.706	0.716	0.442
D19, D20	BERT	**0.794**	**0.886**	**0.793**	**0.793**	**0.793**	**0.589**
M19, M20S	TF-IDF LGBM	0.736	0.831	0.758	0.693	0.724	0.473
M19, M20S	SenLib LGBM	0.683	0.772	0.684	0.681	0.683	0.367
M19, M20S	BERT	**0.809**	**0.902**	**0.826**	**0.784**	**0.804**	**0.619**

## Data Availability

The datasets generated for and analyzed in the present study are available from the corresponding author on reasonable request.
